# Characteristics of Waste Concrete Powder-Based Artificial Fine Aggregate and Its Application in Concrete

**DOI:** 10.3390/ma19040690

**Published:** 2026-02-11

**Authors:** Wei Xu, Liang Zhan, Yang Lei, Lei Xue, Yuguang Zhao, Jun Zhao, Qianyi Zhao

**Affiliations:** 1 China Construction Fifth Engineering Bureau Co., Ltd., Changsha 410004, China; 2 School of Civil Engineering, Zhengzhou University, Zhengzhou 450001, China

**Keywords:** waste concrete powder, artificial fine aggregate, microstructure, mechanical properties, sustainable concrete

## Abstract

Waste concrete powder (WCP), characterized by low reactivity and limited utilization potential, is rapidly accumulating due to the increasing volume of demolition and recycling activities, creating significant environmental and resource challenges. Meanwhile, the shortage of natural fine aggregate (NFA) has become increasingly severe. To address these issues, this study develops a sustainable approach that utilizes WCP as the main raw material, together with fly ash (FA), ground granulated blast-furnace slag (GGBFS), ordinary Portland cement (OPC), and sulphoaluminate cement (SAC), to produce a WCP-based artificial fine aggregate (WAFA) through a cold-bonding process. The physical, mechanical, and microstructural properties of WAFA were systematically analyzed, and its concrete performance was evaluated by replacing NFA at 100% volume. The results show that WAFA exhibits a regular spherical morphology and, after grading adjustment, meets the Zone II sand requirements of GB/T 14684-2022. Increasing the cement content from 2% to 10% raises the 28-day single-particle compressive strength (SPCS) from 12.98 MPa to 23.08 MPa (a 77.8% increase), while enhancing WCP reactivity improves SPCS from 16.17 MPa to 22.80 MPa (a 29.1% increase). Higher cement content and WCP reactivity also promote the formation of C–S–H gel and ettringite (AFt), resulting in higher bulk density, reduced water absorption, and a denser microstructure. In concrete applications, WAFA substantially improves workability, with slump values exceeding those of NFA and recycled fine aggregate (RFA) concretes. Although WAFA concrete exhibits slightly lower compressive and splitting tensile strengths compared with NFA concrete, optimized mix design allows the achievement of target strength grades from C30 to C50, with the C50-W10-50 mixture showing the most favorable mechanical performance. In summary, WAFA shows potential for contributing to the high-value utilization of construction waste and the reduction in natural sand consumption.

## 1. Introduction

With the accelerating pace of global urbanization, the rapid development of the construction industry has not only fueled economic growth but also resulted in the generation of a substantial amount of construction and demolition waste (CDW) [1]. According to statistics, China generated approximately 2.41 billion tons of CDW in 2024 [2]. Among them, waste concrete, as the primary component of CDW, accounts for more than 50% [3]. However, its overall recycling rate was below 10% [4], highlighting the urgent need for efficient recovery and reuse to support the green and low-carbon transition of the construction sector. In the context of the escalating climate emergency, the environmental implications of material consumption and waste generation have become increasingly critical. As noted by Professor Pierrehumbert from Oxford, “With regard to the climate crisis, yes, it’s time to panic” [5], underscoring the necessity for immediate action to mitigate carbon emissions and reduce ecological burdens. The recycling of construction waste, particularly waste concrete, therefore represents not only a resource efficiency strategy but also an essential component of broader climate-oriented sustainability efforts. At present, waste concrete is primarily handled through dumping or disposal in landfills [6]. In fact, a portion of waste concrete is not directly landfilled but is instead processed through crushing, screening, and other procedures to produce recycled coarse and fine aggregates for the preparation of recycled concrete [7,8,9]. However, throughout construction activities, the replacement of old structures, and the production of recycled aggregates, a significant quantity of waste concrete powder (WCP) with particle sizes below 0.15 mm is simultaneously produced [10,11]. Due to its inherently low reactivity and high water absorption, WCP has limited potential for reuse and is commonly disposed of by landfilling or open-air dumping [10,11,12]. Such practices not only occupy substantial land resources but also contribute to dust pollution and impose additional ecological burdens. Consequently, the development of effective treatment methods and high-value recycling technologies for WCP has become an urgent priority to improve resource-utilization efficiency, reduce environmental damage, and contribute to climate-related sustainability goals.

On the other hand, natural fine aggregate (NFA) resources, which are indispensable for concrete production, are experiencing an increasingly critical shortage [13]. Prolonged overexploitation of river sand has resulted in irreversible degradation of riverine ecosystems and severe disturbances to the adjacent geological environment [13,14]. Meanwhile, with the implementation of relevant national policies [14], many sand and gravel plants have been forced to suspend operations or undergo restructuring, leading to a sharp increase in aggregate prices. Consequently, the scarcity of construction sand and gravel has become a major concern. To alleviate the shortage of NFAs, the development of novel and sustainable fine aggregates to replace NFA in concrete production has become a key research focus in the field of concrete materials.

The main component of WCP is hydrated cement mortar, which contains a low proportion of reactive substances after crushing and is therefore classified as a low-activity solid waste [12,15]. Compared with highly active supplementary cementitious materials such as fly ash (FA) and ground granulated blast-furnace slag (GGBFS), which are rich in amorphous SiO_2_ and Al_2_O_3_ [16,17], WCP exhibits a much weaker pozzolanic effect, making its efficient utilization a significant challenge. This challenge has been corroborated by previous research. Xiao et al. [18] demonstrated that the replacement rate of recycled powder in concrete should be maintained between 15% and 30%, as excessive addition can adversely affect workability and increase the risk of early cracking. Jiang et al. [19] found that the compressive strength of artificial coarse aggregates produced from WCP was only 3 MPa, which restricts their use in high-strength construction materials. Similarly, Liu et al. [20] reported that the limited amounts of reactive SiO_2_ and Al_2_O_3_ in WCP hinder the formation of a continuous and compact cementitious network when WCP is used independently as a binder. To address this challenge, an intriguing approach involves incorporating a small amount of highly active cementitious materials—such as ordinary Portland cement (OPC), sulphoaluminate cement (SAC), GGBFA, FA, or alkali activators—to modify the surface or microstructure of low-reactivity WCP. Through such treatment, WCP can be transformed into a fine aggregate, thereby not only overcoming the limitations of WCP utilization but also alleviating the shortage of NFAs.

In recent years, the use of industrial by-products and construction waste for the production of artificial aggregates has attracted increasing attention [20]. Artificial aggregates are generally produced by transforming powder materials into particles with defined shapes and sufficient strength through granulation and hardening processes. Common preparation methods for artificial aggregates include sintering, steam curing, carbonation, and cold-bonding techniques [21,22,23]. Among these methods, the cold-bonding process has shown great application potential due to its low energy consumption, simple procedure, and environmental friendliness [24,25]. To date, various types of cold-bonded artificial fine aggregates (AFAs) have been developed based on the pozzolanic activity of solid waste powders, the cementitious hydration of cement, and the principle of alkali activation. Wu et al. [26] prepared high-sulfur artificial aggregates using GGBFS, steel slag powder, and phosphogypsum as raw materials and found that the inclusion of steel slag powder increased the alkalinity of the system, thereby stabilizing and promoting the formation of C–S–H gel and ettringite (AFt). Dong et al. [27] used FA as the precursor and a composite solution of water glass and sodium hydroxide as the alkali activator. The results showed that when the alkali content (Na_2_O equivalent) was below 5.5%, the single-particle compressive strength (SPCS) increased with the alkali dosage; however, when it exceeded 5.5%, the strengthening effect gradually diminished. Ding et al. [28] produced artificial lightweight fine aggregates using municipal solid waste incineration bottom ash combined with cement and found that a mixture containing 6% FA and 10% silica fume (SF) improved workability and bonding strength by 26.4%. Zhang et al. [29] developed low-carbon and economical high-strength artificial aggregates using gold tailings as the primary raw material for producing lightweight aggregate concrete. The results showed that through hydration and pozzolanic reactions, fluorine gypsum (FG), gold tailings (GT), and sulfur–aluminum–ferric (SAF) cementitious materials mainly formed AFt and C-S-H gel. However, there is still a lack of systematic studies on the preparation of AFAs and their concrete using WCP as the main raw material, combined with highly active cementitious materials such as GGBFS, FA, and a small amount of cement, through a cold bonding process.

Based on this, this study proposes using WCP as the primary raw material, incorporating small amounts of active materials such as cement, FA, and GGBFS, and adopting a cold-bonding process to produce a new type of WCP-based artificial fine aggregate (WAFA). The mix proportions, mechanical properties, physical characteristics, and microstructural features of WAFAs were systematically investigated in order to clarify how cement content and WCP reactivity jointly influence aggregate formation and performance. In addition, WAFAs were applied to concrete as a full (100% by volume) replacement for NFA, and their effects on concrete workability and basic mechanical performance were comprehensively assessed. This study aims to provide new scientific insight into the utilization mechanisms of low-reactivity WCP within multi-component cold-bonding systems, while also offering a feasible technological pathway for the resource utilization of WCP and a potential material alternative to alleviate the shortage of NFAs.

## 2. Materials and Methods

### 2.1. Production of WAFA

#### 2.1.1. Raw Materials of WAFAs

**Figure 1:**
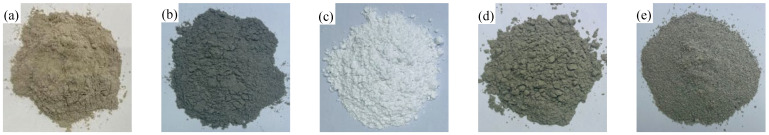
Photographs of raw materials: (**a**) WCP; (**b**) FA; (**c**) GGBFS; (**d**) OPC; (**e**) SAC.

**Figure 2:**
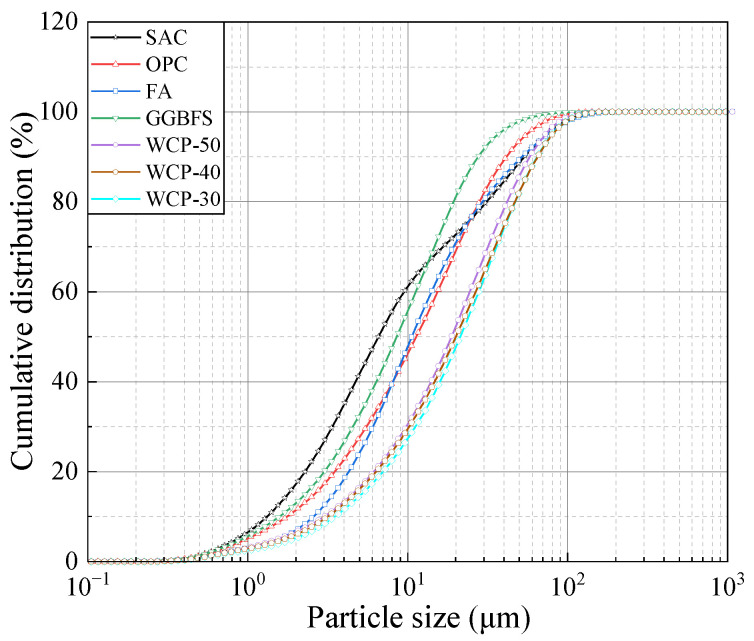
Particle size distribution of the raw materials.

**Figure 3:**
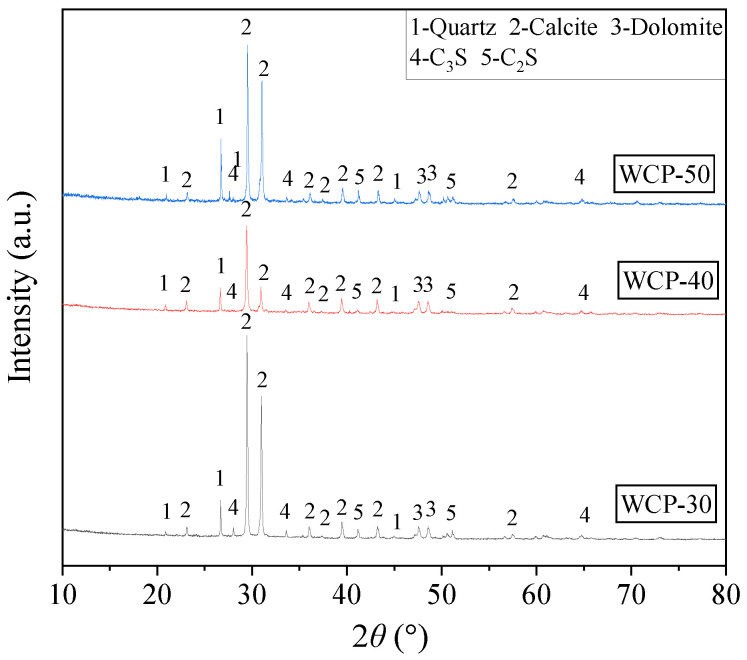
XRD pattern of WCP.

The WAFAs was fabricated using WCP, FA, GGBFS, OPC, and SAC as raw materials, as illustrated in Figure 1. The WCP includes three types (WCP-30, WCP-40, and WCP-50), which were obtained by crushing and screening shear wall specimens with target compressive strength grades of C30, C40, and C50 after testing. Among them, the C30 and C50 shear wall specimens were from the same batch and had been stored for 2 months, while the C40 specimens had been stored for 15 months. The FA used in this study was classified as Class F, Grade I, while the GGBFS was of S95 grade, supplied by Shunsheng New Environmental Building Materials Co., Ltd., Jincheng, China, and conformed to the specifications of ASTM C618 [30]. The OPC used in this study was P.O 42.5 grade, supplied by Tianrui Group Cement Co., Ltd., Zhengzhou, China. The SAC with a strength grade of 42.5 was obtained from Jianwen Special Material Technology Co., Ltd., Zhengzhou, China. Both materials met the specifications of the Chinese standard GB 175-2023 [31]. The particle size distribution of the raw materials was measured using a BT-9300HT laser particle size analyzer (Bettersize Instruments Ltd., Dandong, China), and the results are shown in Figure 2. The chemical compositions of the materials were determined using a Zetium X-ray fluorescence (XRF) spectrometer (PANalytical, Almelo, The Netherlands), and the results are summarized in Table 1. Furthermore, the X-ray diffraction (XRD) patterns of WCP, measured using a SmartLab SE diffractometer (Rigaku, Tokyo, Japan), are shown in Figure 3. The results reveal that the WCP reactivity decreases in the order of WCP-50 > WCP-30 > WCP-40. This phenomenon may be explained by the relatively shorter storage time of the C30 and C50 shear wall specimens, whereas the prolonged storage of the C40 specimens led to nearly complete hydration of the cement particles into C-S-H, resulting in the absence of detectable calcium silicate crystalline phases.

For comparative analysis, both NFA and recycled fine aggregate (RFA) were introduced in this study. The NFA was natural river sand with a fineness modulus of 2.89, complying with the Chinese standard GB/T 14685-2022 [32]. The RFA, referred to as RFA-50, was derived from the crushing and processing of C50-grade shear wall specimens from the same demolition batch as the WCP. It had a particle size range of 0–4.75 mm and satisfied the requirements of the Chinese standard GB/T 25176-2010 [33].

#### 2.1.2. Mix Design

The mix proportions of WAFAs are shown in Table 2. The experiment involved two variables: the type of WCP and the cement content. Three types of WCP were used (WCP-30, WCP-40, and WCP-50), with each constituting 60% of its respective mixture. The cement contents designed in four groups were 0%, 2%, 6% and 10%, with an OPC:SAC ratio of 4:1. In addition, the remaining cementitious materials had an FA:GGBFS ratio of 2:1. The water-to-solid ratio (W/S) was determined through extensive preliminary trials, and the optimal value was set at 0.21.

#### 2.1.3. WAFAs Production

Through multiple comparative tests evaluating the granulation state of WAFA, the best preparation procedure was established as follows: the weighed raw materials were sequentially added into the granulation drum and dry-mixed for 2 min to achieve uniform distribution. Then, the pre-measured water was sprayed into the drum for 1 min while simultaneously increasing the rotational speeds of the mixing blades and the granulation drum. The speeds were maintained until mixing was completed, with a total duration of 25 min. After granulation, the pellets were left to rest for 24 h and then subjected to water-sprinkling curing at room temperature. During the first 7 days, the pellets were sprinkled with water 3–4 times per day; thereafter, sprinkling was performed once every two days until 28 days. Based on extensive preliminary trials, the optimal granulation parameters for WAFA were determined as follows: a total dry raw material mass of 30 kg, a granulator inclination angle of 40°, a granulation-drum rotational speed of 12 rpm, and a mixing-blade rotational speed of 20 rpm. Under these conditions, WAFA pellets with particle sizes in the range of 0–4.75 mm were produced. The aggregates were subsequently sieved and recombined to obtain the target gradation prior to testing.

#### 2.1.4. Test Methods of WAFAs

(1) Single particle compressive strength (SPCS)

At curing ages of 3 and 28 days, ten particles with an average diameter of approximately 2 mm were selected to determine the SPCS of each WAFA sample. Outliers were removed, and the mean value was adopted as the final result. The loading rate of the testing machine was maintained at 0.6 mm/min. The SPCS was calculated according to the following Equation (1) [9,34,35].

(1) $$\sigma =2.8P/\left(\pi d^{2}\right)$$

where *σ* is the single particle compressive strength (MPa); *P* is the load at failure (N); *d* is the average value of diameters, which was measured from three axial dimensions (mm).

(2) Physical properties

The physical properties of WAFAs after 28 days of curing were tested. In accordance with the Chinese standard GB/T 14684-2022 [36], tests were conducted to determine the fineness modulus, particle size distribution, saturated surface-dry water absorption, bulk density, porosity, and apparent density of WAFA. The results were then compared with those of NFAs and RFAs.

(3) Microstructural analyses

The micro-morphology of WAFA was characterized using scanning electron microscopy (SEM), and the phase composition of different WAFA samples was analyzed using XRD.

1. SEM. The micro-morphology of WAFA samples was examined using a desktop SEM (Model: JCM-6000PLUS, JEOL Ltd., Tokyo, Japan), with magnifications ranging from 10× to 60,000×. The test was carried out at an accelerating voltage of 5 kV under high-vacuum mode (≈10^−3^ Pa). To avoid charging effects caused by the insulating nature of the samples, the specimens were gold-sputter coated prior to SEM observation (with a coating thickness of approximately 10 nm).
2. XRD. XRD measurements were performed using Cu Kα radiation (λ = 1.54 Å). Data were collected over a 2θ range of 5–80° with a step size of 0.02°, at a scanning rate of 5°/min. The instrument was operated at 40 kV and 40 mA.

### 2.2. Production of Concrete

#### 2.2.1. Concrete Preparation and Mix Proportion Design

In this study, three strength grades of concrete were designed, namely C30, C40, and C50. Five types of fine aggregates were employed: W2-50, W6-50, W10-50, RFA-50, and NFA. Among them, concretes prepared with RFA-50 and NFA were used as control groups. The raw materials for concrete production included OPC, coarse aggregate (continuously graded gravel with a particle size range of 5–20 mm, complying with the requirements of the Chinese standard GB/T 14685-2022 [32]), fine aggregates (WAFAs, NFAs, and RFAs), FA, water-reducing admixture (WRA), and water. The mix proportion of the NFA concrete was designed according to the Chinese specification JGJ 55-2011 [37]. To maximize the conservation of NFAs, RFAs and WAFAs were used to replace NFAs at a 100% substitution level in the concrete mixtures. Their required quantities were calculated based on an equal-volume replacement method using the NFA dosage as the reference. After determining the amounts of WAFAs and RFAs, additional mixing water was supplied to compensate for their higher water absorption compared with NFAs, the calculation formula is based on Reference [38]. Consequently, the total water-to-binder ratio (w/b) of these mixtures became higher than that of the NFAs reference group. After multiple trial mixes, the final mix proportions of all concretes were determined and are summarized in Table 3.

#### 2.2.2. Experimental Tests on Concretes

The slump test was performed in accordance with the Chinese standard GB/T 50080-2016 [39] to evaluate the workability of concretes incorporating different fine aggregates. In total, 48 cubic specimens (100 mm × 100 mm × 100 mm) and 24 prismatic specimens (100 mm × 100 mm × 400 mm) were cast using the various fine aggregates. Following the procedures specified in GB/T 50081-2019 [40], three specimens from each group were tested at a curing age of 28 days to determine their compressive strength and splitting tensile strength. The experimental investigation focused on elucidating the effects of fine aggregate type on the workability and mechanical performance of concrete.

## 3. Results and Discussion

### 3.1. Analysis of Test Results of WAFAs

#### 3.1.1. Morphology, Gradation, and Fineness Modulus

**Figure 4:**
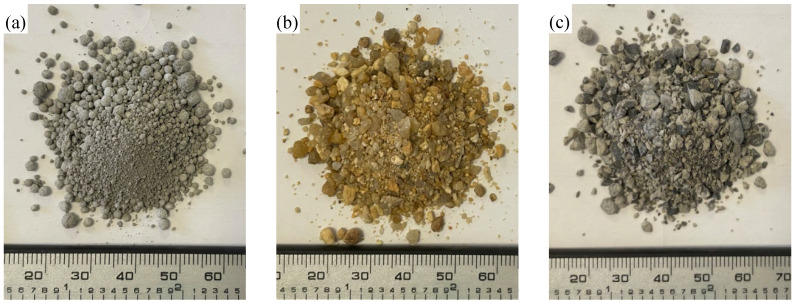
Surface morphology of fine aggregates: (**a**) WAFAs; (**b**) NFAs; (**c**) RFAs.

Figure 4 presents the surface morphology of the three types of fine aggregates: WAFAs, NFAs, and RFAs. The NFAs exhibit relatively smooth surfaces with a distinct granular texture and minimal angularity. In contrast, the RFAs possess irregular shapes with sharp and well-defined edges. The WAFAs, on the other hand, display a predominantly spherical and uniform morphology, which is advantageous for enhancing the workability of concrete mixtures. However, this spherical characteristic may reduce the mechanical interlocking between the fine aggregates and the cementitious matrix, consequently leading to a decrease in the overall strength of the concrete. This is consistent with the conclusion reported by Zhao et al. [9], who found that spherical-shaped aggregates tend to result in lower concrete strength.

**Figure 5:**
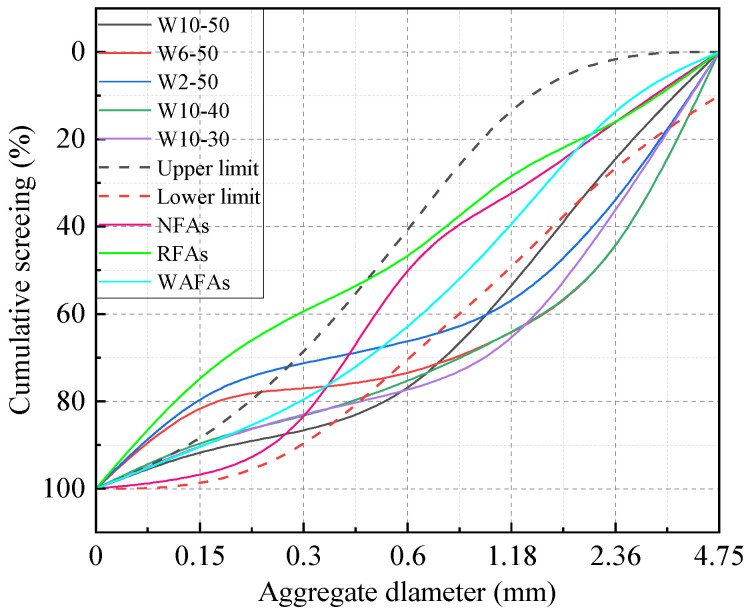
Particle size grading curves.

Figure 5 illustrates the particle size distribution curves of NFAs, RFAs, unadjusted WAFAs, and adjusted WAFAs, while the corresponding sieve pass rates and fineness moduli are summarized in Table 4. The results demonstrate that, after gradation adjustment, the WAFAs satisfy the requirements for Zone II medium sand specified in the Chinese standard GB/T 14684-2022 [36]. As shown in Table 4, significant variations are observed in the bottom-sieve residues of the unadjusted WAFAs. Among the W10-50, W6-50, and W2-50 groups, the W10-50 samples exhibit the lowest residue, indicating that a reduction in cement content during WAFA preparation diminishes the agglomeration ability of the raw powder materials, thereby increasing the proportion of ungranulated fines. Additionally, comparison among the W10-50, W10-40, and W10-30 groups reveals that the source of the parent concrete also influences the fineness characteristics. As the reactivity of the WCP from different parent concretes decreases, the proportion of bottom-sieve residue tends to increase, albeit at a relatively low rate. After gradation adjustment, the particle size distribution and fineness modulus of the WAFAs closely resemble those of the NFAs and are markedly superior to those of the RFAs. This finding suggests that WAFAs possess favorable gradation characteristics and are suitable for application in concrete production.

#### 3.1.2. Bulking Density, Apparent Density, and Water Absorption of Saturated Surface-Dry Condition

**Figure 6:**
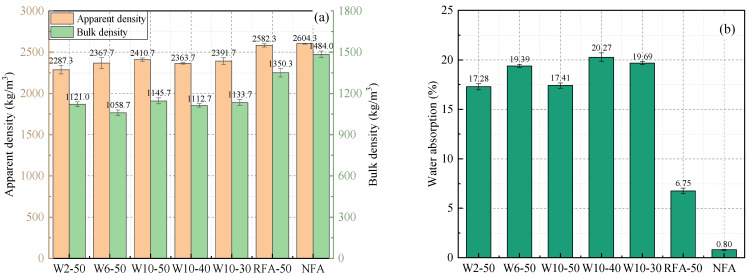
Physical properties of fine aggregates: (**a**) Apparent density and bulking density; (**b**) Saturated surface-dry water absorption.

Figure 6 illustrates the bulking density, apparent density, and saturated surface-dry water absorption of the different fine aggregates. The NFAs exhibit the highest bulking and apparent densities, accompanied by the lowest water absorption, which can be attributed to their dense and compact natural structure. In contrast, both RFAs and WAFAs display relatively lower apparent and bulking densities but markedly higher water absorption values. This phenomenon is primarily associated with the loose and porous microstructures of these recycled and waste-derived fine aggregates.

For WAFAs with varying cement contents (W2-50, W6-50, and W10-50), the apparent density exhibits an overall increasing trend with higher cement content, whereas the bulking density first decreases and then increases, and the water absorption initially rises before subsequently declining. This behavior can be attributed to the influence of cement content on particle bonding and microstructure formation. When the cement content is 2%, the W2-50 sample exhibits weak cohesion, resulting in a greater amount of free fine powder. These fine particles fill the interstitial voids between aggregate grains, thereby enhancing the overall packing compactness and yielding a relatively high bulking density. The lower water absorption of W2-50 compared with W6-50 and W10-50 may be due to its excessively loose internal structure, which contains a large number of interconnected pores. During the measurement of saturated surface-dry water absorption, part of the internal moisture may evaporate prematurely through these open pores, leading to an underestimation of the measured absorption value. In contrast, the trend observed from W6-50 to W10-50 aligns more closely with the expected behavior. A moderate increase in cement content enhances the interfacial structure between WCP particles [41], effectively filling microcracks and pores. This improvement increases the compactness of the WAFAs, resulting in higher density and reduced water absorption. This densification-driven “density up–absorption down” trend has been widely reported for cold-bonded/artificial aggregates produced from construction and demolition waste powders or blended binders (e.g., recycled concrete powder–based aggregates and demolition-derived pellets), where enhanced bonding and gel formation densify the granules, increasing density while reducing water absorption [42,43,44]. Similar trends are also reported in geopolymer/cold-bonded lightweight aggregate systems, in which increasing the degree of densification (via binder composition or activator/binder adjustment) is accompanied by reduced water absorption and enhanced compactness [45].

On the other hand, under the same cement content, the WAFAs prepared from different sources of WCP (W10-30, W10-40, and W10-50) exhibited significant differences. Among them, W10-50 showed higher density and lower water absorption, whereas W10-40 exhibited the lowest density and the highest water absorption. This trend is consistent with the sequence of active component contents determined for the corresponding WCPs in Section 2.1.1, suggesting that the activity level of WCP has a significant effect on the physical characteristics of WAFAs. Highly active WCP particles are denser and generate more abundant hydration products, thereby forming a more compact skeletal structure and enhancing the overall quality and performance of the WAFAs.

#### 3.1.3. Single Particle Compressive Strength (SPCS)

**Figure 7:**
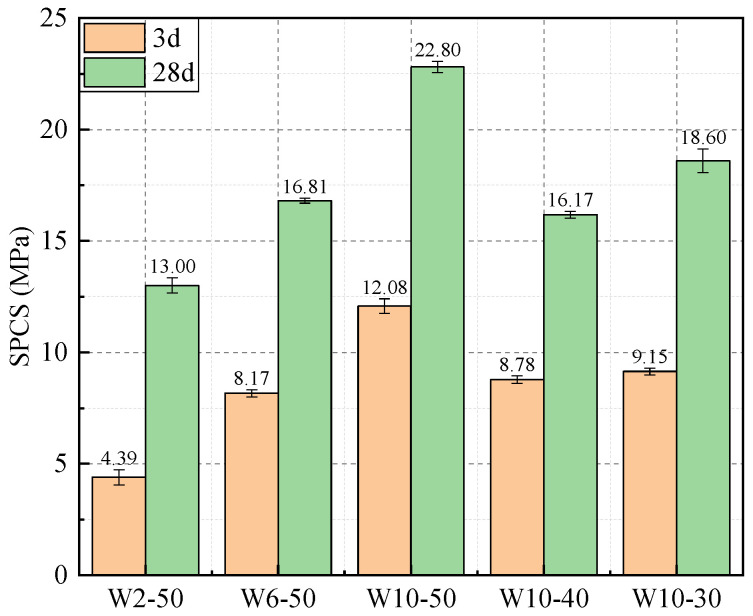
SPCSs of WAFAs.

Figure 7 presents the SPCSs of the WAFAs. Noticeable differences in SPCS were observed among WAFAs with different mix proportions. For WAFAs with varying cement contents (W2-50, W6-50, W10-50), the 28-day SPCS increases from 13.00 MPa (W2-50) to 22.80 MPa (W10-50), a 75.4% gain. This improvement can be attributed to the role of cement during the cold-bonding process, where it not only provides initial cementation but also generates Ca(OH)_2_, C-S-H gel, and a small amount of Aft at early ages [46]. In addition, the alkaline environment established by cement hydration promotes the pozzolanic reactions of FA and GGBFS, as well as the potential hydration of WCP, forming additional C-(A)-S-H gel [47,48]. These hydrates bind WCP particles into a dense shell, while unreacted FA and GGBFS contribute a secondary micro-filling effect, further densifying the WAFAs. Moreover, the combined use of OPC and SAC exhibits a synergistic effect: the rapid early reaction of SAC can synergistically promote the formation of C-S-H gel with OPC [49], while the generation of Aft crystals further enhances the compactness of WAFA. Consequently, as the cement content increases, the 3-day and 28-day SPCS values of WAFA are significantly improved.

At the same cement content (10%), the SPCSs of WAFAs (W10-30, W10-40, and W10-50) prepared from different sources of WCP exhibited significant differences, following the order W10-50 > W10-30 > W10-40. These variations are primarily attributed to differences in the reactivity of the WCPs. WCP-50 contains a greater proportion of partially unhydrated cement clinker phases (such as C_2_S and C_3_S), which can be reactivated during the cold-bonding process [50]. Upon contact with water, these phases generate C-S-H gels and Ca(OH)_2_, the latter promoting a more alkaline environment. This elevated alkalinity accelerates the secondary hydration reactions of the silicoaluminate glass, soluble SiO_2_, and soluble Al_2_O_3_ in FA and GGBFS, resulting in the formation of additional C-(A)-S-H gels. Consequently, these synergistic reactions lead to the observed differences in the SPCS of WAFAs. In summary, a moderate increase in cement content and the use of highly reactive WCP can substantially improve the single-particle compressive strength of WAFA. This trend is consistent with the general understanding reported in recent state-of-the-art reviews on cold-bonded artificial aggregates, which emphasize that pellet strength is primarily governed by binder-driven hydration formation and the resulting densification of the granule shell and interparticle bonding network [44]. Comparable cement/binder-dose–dependent strength enhancement has also been reported in cold-bonded aggregate systems (e.g., core–shell pellets and multi-solid-waste cold-bonded aggregates), where increasing cementitious binder content promotes the formation of C–S–H-type gels and crystalline hydrates that fill pores and microcracks, leading to higher pellet strength and improved integrity [51,52]. Moreover, the explanation that differences among W10-30, W10-40, and W10-50 originate from WCP reactivity is consistent with recent recycled concrete powder (RCP)–based artificial aggregate studies, which show that higher contents of reactive clinker phases and enhanced alkalinity can increase secondary reactions (including additional gel formation), thereby improving single-particle strength and densifying the microstructure [43]. Overall, the present results fall within the range of mechanisms reported for RCP-derived artificial aggregates, reinforcing that binder dosage and precursor reactivity jointly regulate hydrate formation, shell densification, and ultimately the SPCS of cold-bonded aggregates [43,44].

#### 3.1.4. X-Ray Diffraction (XRD) Analysis

**Figure 8:**
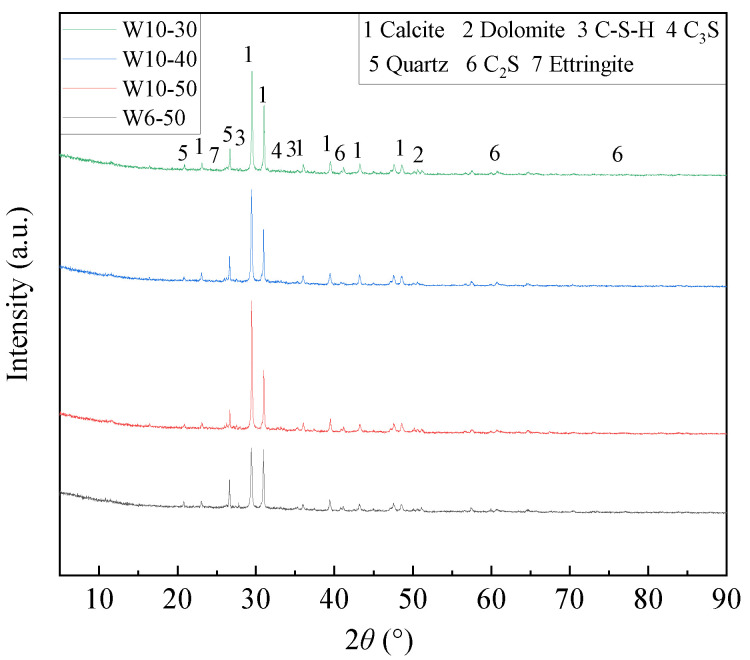
XRD Analysis of WAFAs.

Figure 8 presents the XRD analysis results of different WAFAs. The main mineral phases of different WAFAs include Calcite (PDF#05-0586), Quartz (PDF#46-1045), and Dolomite (PDF#20-0452) [53], along with partially hydrated phases such as C-S-H (PDF#33-0306) and Ettringite (PDF#41-1451). Specifically, WAFAs with different cement contents (W6-50 and W10-50) both exhibit a prominent calcite diffraction peak at 2θ ≈ 29.4° and a broad C-S-H diffraction band within the 2θ = 25~35° range. However, the calcite peak intensity of W10-50 is significantly higher than that of W6-50, and more C-S-H phases are detected. This indicates that an increased cement dosage enhances the early release of Ca(OH)_2_ (CH), which reacts with the active SiO_2_ and Al_2_O_3_ in FA and GGBFS to form C-S-H and C-A-S-H gels [48]. The remaining CH subsequently reacts with atmospheric CO_2_ to produce CaCO_3_, thereby improving the overall cementitious degree and structural compactness of the system. Meanwhile, both W6-50 and W10-50 exhibit weak AFt peaks, confirming that a small amount of AFt is formed through the early reaction of SAC with gypsum.

For WAFAs with the same cement content (W10-50, W10-40, and W10-30), distinct calcite diffraction peaks and C-S-H phases were also detected, with the intensity of the calcite peaks following the order W10-50 > W10-30 > W10-40. This trend corresponds to the WCP reactivity sequence described in Section 2.1.1 and is strongly associated with the residual unhydrated C_3_S (PDF#16-0407) and C_2_S (PDF#33-0303) contents in the WCP. The unhydrated C_3_S and C_2_S phases are reactivated during the cold-bonding process, participating in hydration to produce CH and C-S-H gels. Among these, WCP-50 contains the highest proportion of unhydrated C_3_S and C_2_S, resulting in more extensive formation of CH and C-S-H gels. During the cold-bonding process, the surface CH undergoes mild carbonation, thereby promoting the increased formation of CaCO_3_ in W10-50.

The XRD analysis demonstrates that both the cement content and the reactivity of the WCP jointly determine the mineral composition and the extent of hydration reactions in WAFA. A higher cement content combined with highly active waste concrete powder promotes the formation of C-S-H phases and Calcite, thereby achieving a synergistic enhancement in the structural densification and mechanical performance of the artificial fine aggregates.

#### 3.1.5. Microstructure Analysis

**Figure 9:**
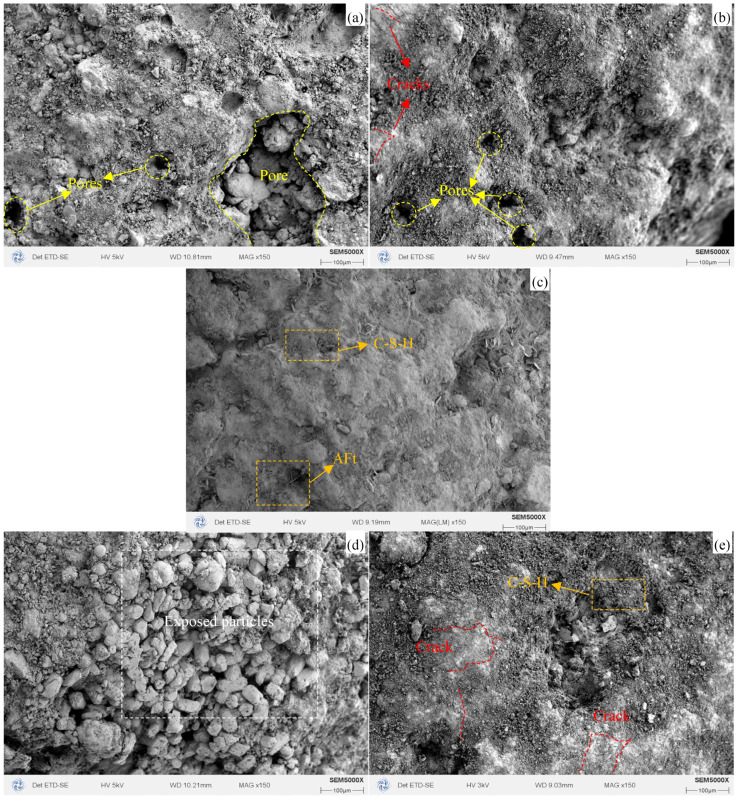
SEM Images of WAFAs: (**a**) W10-30; (**b**) W10-40; (**c**) W10-50; (**d**) W0-50; (**e**) W6-50.

Figure 9 presents the SEM analysis results of W10-50, W10-40, W10-30, W6-50, and W0-50. It can be observed that W10-50 exhibits a more complete hydration process, with abundant hydration products such as C-S-H gels and AFt. The aggregate surface shows no visible pores or microcracks, which is one of the reasons for its superior physical and mechanical properties. In contrast, W10-40, W10-30, W6-50, and W0-50 exhibit a lower degree of hydration, resulting in fewer hydration products and limited gel-filling effects. Consequently, numerous pores and microcracks are present in these samples. Particularly for W0-50, almost no cementitious products are observed in the microstructure, and the particles appear loosely packed with many exposed surfaces. These differences are mainly attributed to the higher reactivity of WCP-50 compared with WCP-40 and WCP-30, which leads to the generation of more hydration products and a denser microstructure in W10-50. Moreover, due to its higher cement content compared with W6-50 and W0-50, W10-50 produces a larger quantity of hydration products, effectively refining its internal structure and enhancing overall compactness.

### 3.2. Concrete Performance Analysis of WAFAs

#### 3.2.1. Slump

**Figure 10:**
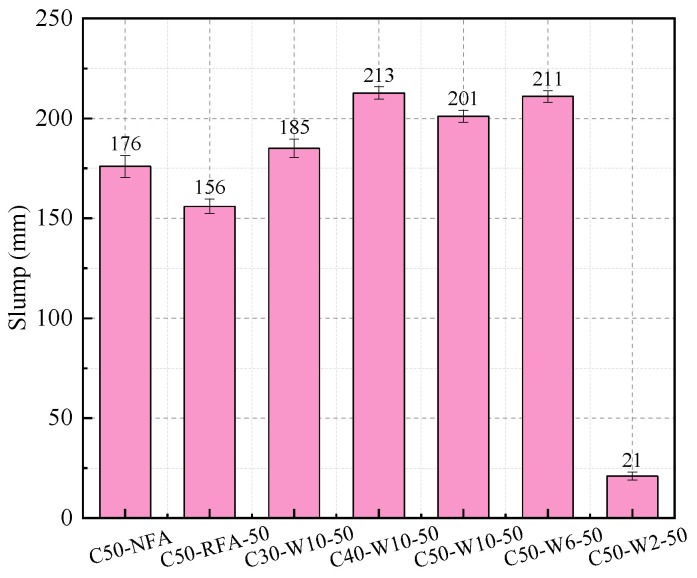
Slump of concrete.

Figure 10 shows the slump test results of concretes prepared with different fine aggregates. It can be observed that the most exceptional case is C50-W2-50, which exhibited almost no slump. This finding supports the inference made in Section 3.1.2 that W2-50 contains numerous interconnected pores, leading to an underestimated saturated surface-dry water absorption during testing. Consequently, the additional water content calculated for C50-W2-50 during casting was inaccurate, resulting in nearly no workability; therefore, W2-50 was excluded from subsequent concrete casting. After removing the abnormal slump value of C50-W2-50, the workability of the remaining WAFA concretes was found to be relatively consistent. Compared with concretes made using other fine aggregates of the same strength grade, the slump values of WAFA concretes were all higher than those of NFA- and RFA-based concretes, among which C50-RFA-50 exhibited the lowest slump. This can be attributed to the spherical morphology of WAFA, which effectively reduces the friction between fine and coarse aggregates as well as among fine aggregate particles [9]. In addition, during mixing, the WAFA had not yet fully absorbed the additional water, allowing part of this water to act as mixing water, thereby improving concrete workability. In contrast, in C50-RFA-50, the angular shape of RFA-50 increases interparticle friction. Moreover, as shown in Table 4, particles smaller than 0.15 mm account for approximately 40% of RFA, and these fine powders adhere to the aggregate surface, increasing both the specific surface area and paste viscosity, which ultimately results in the lowest slump.

#### 3.2.2. Compressive Failure Mode and Cubic Compressive Strength

**Figure 11:**
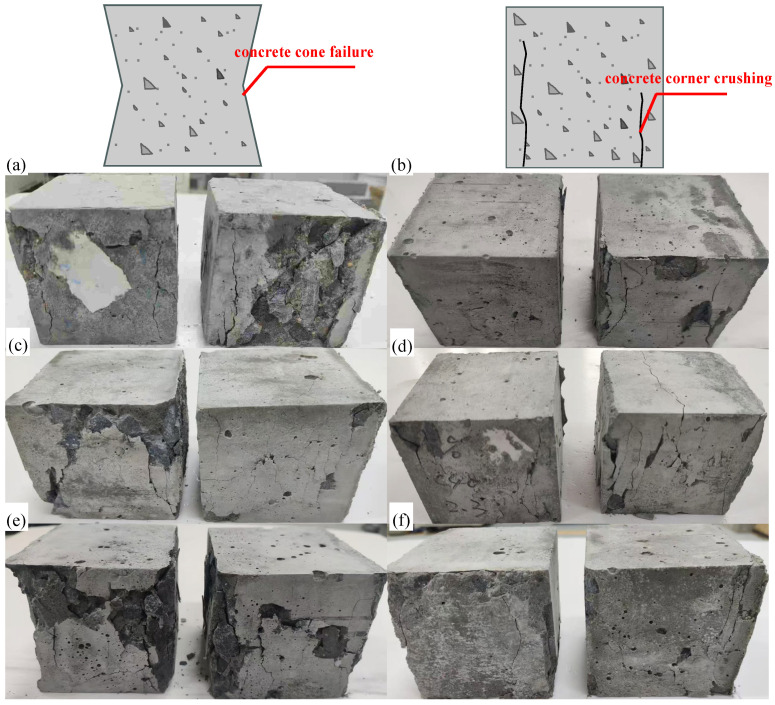
Compressive failure mode: (**a**) C50-NFA; (**b**) C50-RFA-50; (**c**) C30-W10-50; (**d**) C40-W10-50; (**e**) C50-W10-50; (**f**) C50-W6-50.

Figure 11 illustrates the compressive failure modes of cubic specimens prepared with different fine aggregates. Two types of failure modes were observed in total. The first type is a conical failure, where the specimen exhibits lateral expansion in the midsection under compression, leading to surface spalling and the formation of a symmetrical four-cornered conical shape [54]. The second type is a corner failure, characterized by the development of nearly vertical cracks at the specimen corners, which subsequently propagate downward to the base until complete failure occurs [55].

**Figure 12:**
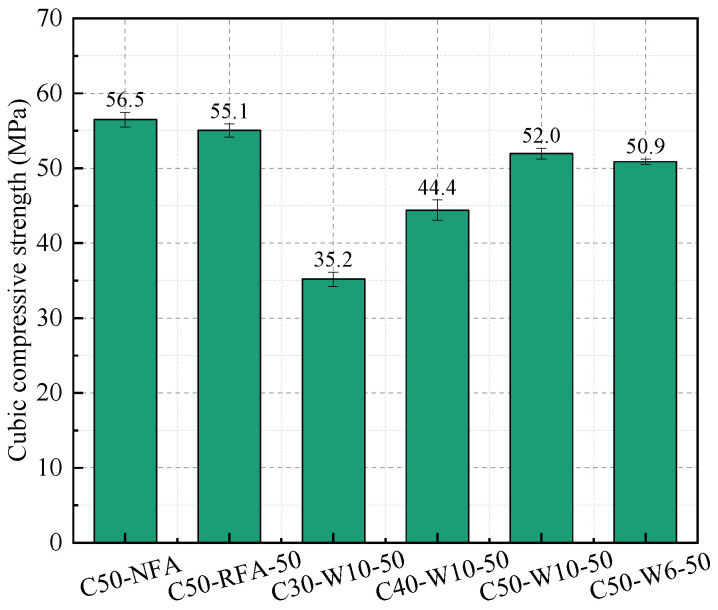
Cubic compressive strength of concrete.

Figure 12 presents the 28-day compressive strength of concrete specimens incorporating different fine aggregates. Compared with NFA and RFA concretes of the same strength grade, WAFA concrete requires significantly more water at a comparable binder content because WAFA has a much higher water absorption than NFA and RFA, necessitating additional water during mixing. When the mix design is properly adjusted, however, concrete incorporating WAFA can still achieve the target strength grade. For mixtures with the same C50 strength grade, the compressive strength follows the order: C50-NFA > C50-RFA-50 > C50-W10-50 > C50-W6-50. This difference can be attributed to the fact that the additional water used during the preparation of WAFA concrete may not have been fully absorbed by the WAFA, resulting in an actual water-to-binder ratio higher than that of NFA and RFA concretes. Moreover, due to the spherical morphology of WAFA, the mechanical interlocking between WAFA particles and the cement paste is slightly weaker than that in C50-NFA and C50-RFA-50, leading to a reduction in compressive strength for WAFA concretes of the same strength grade. In comparison, C50-W10-50 exhibited slightly higher strength than C50-W6-50, primarily because W10-50 contains a higher amount of unhydrated cement and pozzolanic materials than W6-50. These components continue to absorb water and undergo further hydration and pozzolanic reactions, generating larger quantities of C-S-H gel and AFt than in W6-50. Additionally, due to the internal curing effect [34,56], as the WAFA absorbs water, the ongoing hydration of cement consumes moisture and creates a local humidity gradient, enabling the absorbed water within WAFA to be gradually released into the surrounding paste, thus promoting continued hydration and strengthening both the matrix and the interfacial transition zone (ITZ). Another contributing factor is the lower cement content in W6-50, which results in insufficient aggregate strength and reduced hydration, ultimately leading to the higher compressive strength observed in C50-W10-50 compared with C50-W6-50.

#### 3.2.3. Splitting Tensile Strength

**Figure 13:**
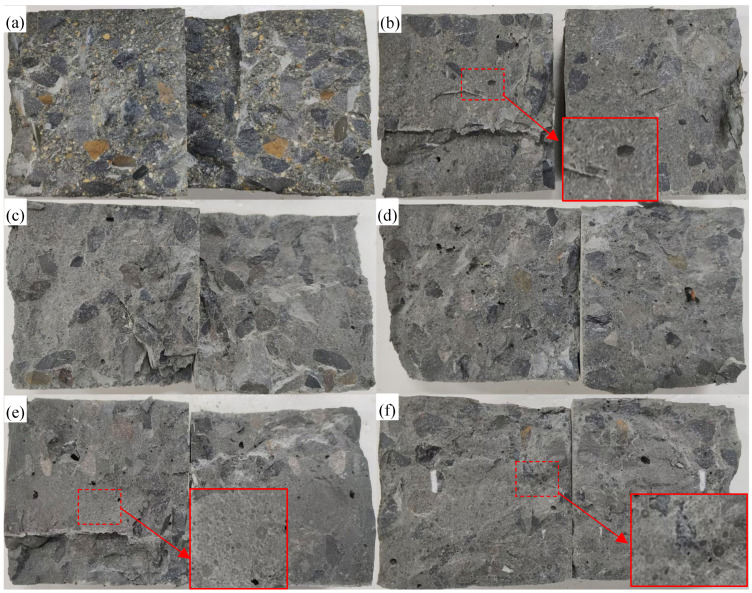
Splitting tensile failure mode: (**a**) C50-NFA; (**b**) C50-RFA-50; (**c**) C30-W10-50; (**d**) C40-W10-50; (**e**) C50-W10-50; (**f**) C50-W6-50.

Figure 13 shows the splitting tensile failure patterns of concrete cubes prepared with different fine aggregates. The fracture surfaces of all concretes are irregular and uneven, with coarse aggregates protruding from the fracture plane and voids formed due to separation between the coarse aggregates and the cement mortar. The splitting failure of each type of concrete originated from cracking within the ITZ. The fracture morphology indicates that most coarse aggregates remained intact, and the cracks predominantly propagated around the aggregates, suggesting that the tensile strength of the coarse aggregates exceeds that of the cement matrix. In the WAFA concretes, numerous spherical WAFA particles were observed to be split into two halves, displaying smooth fracture surfaces with microcracks penetrating through the particle interiors. In contrast, only a few fine aggregate fractures were observed in the RFA concretes, while the NFA concretes showed no noticeable fine aggregate fracture.

**Figure 14:**
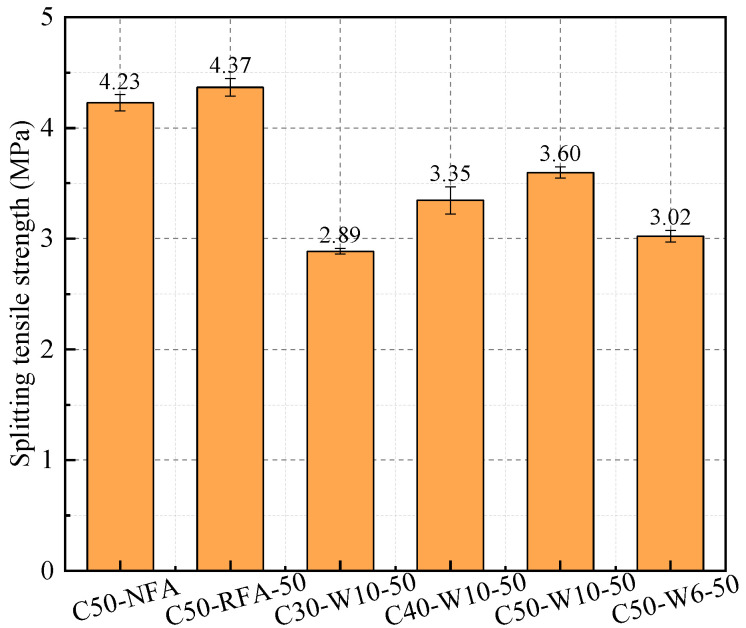
Splitting tensile strength of concrete.

Figure 14 presents the 28-day splitting tensile strength of concretes incorporating different fine aggregates. Distinct variations are observed among the mixtures. For WAFA concretes, the splitting tensile strength of C30-W10-50, C40-W10-50, and C50-W10-50 increases with the designed strength grade. At the same strength level, C50-W6-50 exhibits a significantly lower splitting tensile strength than C50-W10-50. Among the C50 concretes incorporating different fine aggregates (C50-NFA, C50-RFA, and C50-W10-50), the C50-W10-50 mixture shows the lowest splitting tensile strength. The reason for this can be explained as follows: observations of the fracture surfaces show that during failure, the coarse aggregates, which serve as the primary bridges transmitting tensile stress [57], remain intact, while cracking occurs within the ITZ and the surrounding cement mortar [58]. In addition, fine aggregates embedded in the cement matrix also fracture, indicating that the tensile strength of the cement paste is lower than that of the coarse aggregates. Consequently, after debonding occurs at the ITZ, the fine aggregates and the surrounding paste jointly bear the applied tensile stress. Among the different fine aggregates, NFA consists of natural rock particles, RFA comprises rock particles coated with a small amount of old mortar, whereas WAFA is composed of spherical cement-based particles. Owing to their cementitious nature, the tensile strength of WAFA is considerably lower than that of NFA and RFA. As a result, WAFA is more susceptible to fracture under tensile stress. Therefore, at the macroscopic level, the splitting tensile strength of WAFA concrete is significantly lower than that of RFA and NFA concretes of the same strength grade.

## 4. Conclusions

In this study, a novel waste concrete powder-based artificial fine aggregate (WAFA) was successfully fabricated using a cold-bonding process with waste concrete powder (WCP) as the primary raw material, supplemented with fly ash (FA), ground granulated blast furnace slag (GGBFS), ordinary Portland cement (OPC), and sulphoaluminate cement (SAC). The physical and mechanical properties of WAFA, as well as its application performance in concrete, were systematically investigated. The main conclusions are as follows:

1. The WAFA consists of 60% WCP, 10–13.3% GGBFS, and 20–26.7% FA, achieving an overall solid waste utilization rate of 90–100%, which aligns with the objective of high-percentage solid waste elimination.
2. The WAFA particles exhibit a regular spherical shape. After gradation adjustment, the WAFA meets the requirements for Zone II medium sand specified for construction use, thereby possessing the fundamental properties necessary to replace NFA.
3. The physical and mechanical properties of WAFA are jointly governed by the cement content and the reactivity of WCP. Increases in cement content and WCP reactivity both contribute to higher apparent density and lower water absorption. When the cement content increases from 2% to 10%, the 28-day single-particle crushing strength rises from 12.98 MPa to 23.08 MPa, representing a 77.8% improvement. Similarly, as the reactivity of WCP increases, the 28-day single-particle crushing strength increases from 16.17 MPa for W10-40 to 22.80 MPa for W10-50, corresponding to a 29.1% enhancement.
4. The XRD and SEM analyses confirm that a high cement content combined with highly reactive WCP accelerates hydration, leading to the formation of abundant C-S-H gels and AFt. This results in a denser microstructure and enhanced interparticle bonding. In contrast, samples with low cement content or low-reactivity WCP exhibit porous surfaces with visible microcracks. These microscopic differences directly determine the strength and water absorption behavior of WAFA.
5. The workability of WAFA concrete is excellent. The spherical shape of WAFA reduces internal friction between aggregates, resulting in a significantly higher slump compared with concretes made with NFA and RFA.
6. The compressive strengths and splitting tensile strengths of WAFA concrete are slightly lower than those of concretes made with NFA and RFA; however, the reductions are relatively limited in magnitude. Among all mixtures, the C50-W10-50 group shows the best overall performance, achieving the target strength while maintaining high workability and structural integrity.

In summary, as a novel artificial fine aggregate, WAFA exhibits good workability and adequate mechanical performance in concrete. It provides a new pathway for the efficient recycling of construction waste and serves as a promising alternative to NFA. Future research should focus on optimizing mix design and interface enhancement strategies to further improve its potential for use in high-performance concrete. At the same time, it is important to acknowledge the scope and limitations of the present study, particularly the absence of micro-scale characterization (such as ITZ morphology, pore structure distribution, and other microstructural features), which plays a crucial role in understanding how microstructural behavior influences aggregate performance. Therefore, comprehensive microstructural analysis will form a central part of our subsequent research in order to better elucidate these underlying mechanisms.

## Figures and Tables

**Table 1:** Chemical compositions (wt.%).

Raw Materials	SiO_2_	Al_2_O_3_	Fe_2_O_3_	CaO	MgO	SO_3_	K_2_O	TiO_2_	MnO	Na_2_O	Loss
WCP-30	21.11	7.82	3.22	57.16	7.20	1.08	-	0.35	-	0.41	1.65
WCP-40	22.86	7.92	3.51	57.01	5.08	1.20	-	0.32	-	0.32	1.78
WCP-50	22.12	7.06	4.48	56.08	5.51	1.21	-	0.33	-	0.35	2.86
FA	50.30	19.90	6.56	13.30	0.81	1.73	1.60	-	-	2.30	2.02
GGBFS	30.15	14.42	0.44	39.88	9.26	3.28	0.39	1.05	0.50	0.28	0.35
OPC	24.14	5.82	3.22	58.24	4.07	4.48	1.64	-	-	-	1.09
SAC	10.08	17.65	3.13	47.87	1.82	16.54	1.23	0.78	-	0.38	0.52

**Table 2:** The mix proportions of WAFAs.

Notations	Mix Proportions (wt.%)							W/S
	OPC	SAC	GGBFS	FA	WCP-30	WCP-40	WCP-50	
W10-30	8.0	2.0	10.0	20.0	60.0	0	0	0.21
W10-40	8.0	2.0	10.0	20.0	0	60.0	0	0.21
W10-50	8.0	2.0	10.0	20.0	0	0	60.0	0.21
W6-50	4.8	1.2	11.3	22.7	0	0	60.0	0.21
W2-50	1.6	0.4	12.7	25.3	0	0	60.0	0.21
W0-50	0.0	0.0	13.3	26.7	0	0	60.0	0.21

In the specimen labels, W10 denotes WAFA prepared with a 10% cement content, and 30 refers to WCP sourced from crushed shear walls with a compressive strength grade of C30. Accordingly, W10-30 represents WAFA produced using WCP from C30-grade shear walls with a 10% cement dosage. The remaining designations follow the same pattern.

**Table 3:** Mix proportions of concrete (Unit: kg/m^3^).

Concrete Type	Strength Grade	OPC	Coarse Aggregate	Fine Aggregate	FA	Water	WRA	w/b
C30-NFA	C30	294.7	1171.0	710.1	52.0	172.9	0.31	0.50
C40-NFA	C40	334.0	1142.4	692.8	58.9	172.9	0.35	0.44
C50-NFA	C50	363.7	1120.7	679.6	64.2	172.9	0.47	0.40
C50-RFA-50	C50	363.7	1120.7	686.9	64.2	207.9	0.47	0.49
C30-W10-50	C30	294.7	1171.0	654.0	52.0	282.4	0.31	0.81
C40-W10-50	C40	334.0	1142.4	638.0	58.9	279.8	0.35	0.71
C50-W10-50	C50	363.7	1120.7	625.9	64.2	277.7	0.47	0.65
C50-W6-50	C50	363.7	1120.7	618.3	64.2	295.3	0.47	0.69
C50-W2-50	C50	363.7	1120.7	598.2	64.2	271.4	0.47	0.63

**Table 4:** Screening percentages of each grade and fineness modulus of different fine aggregates.

Types	Screening Percentages of Each Grade (%)							Fineness Modulus
	4.75 mm	2.36 mm	1.18 mm	0.6 mm	0.3 mm	0.15 mm	Bottom	
NFAs	0.0	15.70	17.8	11.0	45.0	8.3	2.2	2.81
RFAs	0.0	17.4	8.8	22.5	10.1	13.7	27.5	2.21
W10-50	0.0	23.2	31.3	25.3	7.5	3.5	9.3	3.32
W6-50	0.0	50.1	15.0	9.4	2.9	0.8	21.8	3.44
W2-50	0.0	35.8	23.9	7.0	4.4	5.6	23.3	3.04
W10-40	0.0	50.2	14.4	11.2	8.0	4.7	11.5	3.60
W10-30	0.0	37.0	32.3	8.7	4.6	7.2	10.3	3.57
WAFAs	0.0	10.4	30.1	23.4	16.8	9.8	9.4	2.83

## Data Availability

The original contributions presented in this study are included in the article. Further inquiries can be directed to the corresponding author.
